# Progress in Surgery

**Published:** 1900-03-03

**Authors:** 


### Progress in Surgery.

### SURGICAL DISEASES OF THE KIDNEYS AND URETERS.

**Renal Calculus.**—Dr. Briddon[1] reports a case in which he removed by operation 149 calculi from a kidney. They varied in size from a small hen's egg to a bird shot, and weighed altogether six and a quarter ounces (troy). The largest calculus was four and three-quarter inches by four inches in circumference. The history of the case is briefly as follows: The patient was a woman of 38, and had previously suffered from repeated attacks of left renal colic, and passed small calculi. These attacks had ceased three years, when she was seized with severe renal colic on the right side, with vomiting, but with no hæmaturia or frequency of micturition. The right kidney was felt to be as large as a cocoanut, and tender. The ureters were catheterised, and the two specimens of urine found to have the same character and to contain albumen, hyaline and pus casts, and groups of epithelial cells, showing pyelo-nephritis. Lumbar nephrotomy was performed, the posterior border of the kidney being incised for two inches. Fifty or sixty calculi were then removed, and then more calculi were felt through thin partition walls. There were five or six such cavities. On the twenty-fourth day the patient died from uræmic convulsions and coma. In a discussion in the New York Surgical Society,[2] Dr. P. Syms said that there were cases where the clinical symptoms strongly pointed to the presence of stone in the kidney, but in which X-ray photography gave absolutely negative results. Dr. Abbe replied that if a good X-ray picture, displaying ribs and spine well, and perhaps corroborated by a second one, failed to show the presence of a stone in the kidney, he felt well satisfied there was no stone of any size there. Dr. W. H. Thomson[3] states that whether a stone-bearing kidney should be excised or not depends largely upon the state of the kidney tissue itself. When suppuration has already supervened incision into the organ can be expected to give only temporary relief. Hence early diagnosis was very important, though the difficulties were many in making it. An exploratory operation was warranted whenever there were present polyuria, frequent urination, recurrent hæmaturia, and aching loins, even though pus had not appeared in the urine, and no tumefaction could be felt in the region of the kidney. Dr. P. J. Freyer,[4] in a clinical lecture on the symptoms of stone in kidney, quoted a case in which symptoms had lasted three years, beginning with painless hæmaturia. After some weeks pain was super-added. For a year and a half the pain was in the form of attacks of renal colic, but after this the colic disappeared, and was replaced by a fixed aching pain in the loins. Freyer explained this change of character of the pain by the gradual fixation of the stone in a calyx as it enlarged. There was a tender renal swelling on the right side. The case quoted was remarkable in that there was no pus in the urine; nor was there any mucus. Harrison has pointed out that large quantities of mucus are found even in an early stage of stone in young people. The patient, moreover, had no symptoms referred to the bladder or urethra. Freyer states that traumatism, especially if there is associated effusion of blood into the kidney, is frequently followed by stone. At the operation a stone about the size of an almond was successfully removed.

**Calculus in the Ureter.**—Mr. Henry Morris[4] contributes an exhaustive paper upon this important subject, and tabulates 47 cases which have been operated upon. The following is an abstract of this paper. Vesical calculus is much more frequent in males than females, because the urethra is longer. This difference does not exist in renal and ureteral calculus, and in the table 27 of the 47 cases were in females. Concretions formed primarily in the ureter are very rare. When they occur they are phosphatic, and deposited above a stricture of, or upon an ulcer of, the ureter, or upon a foreign body, such as a catheter *en demeure*. Ureteral stones descend from the kidney, and become impacted by reason of their volume or roughness. As to the duration of impaction it may be for an indefinite time or permanently. Morris has operated upon six cases and found no renal stone, though there must have been a ureteral stone present, and five out of the six subsequently passed stones; one during convalescence, two after about 15 months, and the others after 9, 13, and 5½ months. In each of the latter three cases there is good reason to believe that the kidney afterwards perfectly resumed its function. Thus the impaction may persist for many months, and not destroy the kidney, if the obstruction in the ureter is not complete. The position of the impaction in the ureter is usually at one of the places where normally the ureter has the smallest diameter, and these places are—firstly, at a point about two inches from the hilum of the kidney; secondly, where the ureter enters the wall of the bladder, and especially at its termination; and thirdly, where the ureter crosses the brim of the pelvis, the narrowing at this point being less marked than at the other two. Further, the curved direction of the ureter a little below the rim of the pelvis seems to cause impaction in that situation sometimes. Morris's analysis of 44 operation cases in which the situation of impaction is given shows it to have been in 19 within two inches of the kidney, in 15 just before or where passing through the vesical wall, and in 11 somewhere about the brim of the true pelvis. In one case recorded in the Pathological Society's Transactions the lower opening of the ureter was entirely obliterated, and three calculi were lodged in separate compartments of the ureter, formed one above the other by constricting bands on its inner wall. In some post mortem cases the ureter has been found filled nearly from end to end with calculi.

Being formed in the kidney, the composition of the stones may be of uric acid, urates, calcium oxalate, or phosphates, and they vary much in volume and number. Roberts relates a case in which a single stone, the size of a hemp seed, weighing one and a third grains, and another in which a stone the size of a small pea killed their victims. If stones remain long in the ureter they may increase by deposition of fresh salt from the urine upon them at either extremity. The oxalate calculi are often rough and fixed by their projections into the mucous membrane, and may be difficult to dig out. The precise location of a calculus cannot be made without operation, unless it can be felt through the abdominal parieties, the rectum, the vagina, or the bladder. In all cases of nephro-lithotomy the ureter should be explored with a probe as far as the bladder. As to diagnosis, in many cases lumbar exploration alone can affect it. Stone in the kidney produces much the same symptoms, and often the pain in that disorder is referred to the ureter, or to the bladder, ovary, or testicle. Moreover, renal calculi frequently co-exist with ureteral. In the lower end of the ureter the condition may closely simulate encysted vesical calculus. Calculi in this situation, failing to enter the bladder, may be driven through the side of the ureter, and get lodged between the coats of the bladder towards its neck. Such a cavity may form a communication with the bladder. In both conditions there may be incomplete ureteral obstruction, which occasionally becomes complete, in which cases nephrectasis, *i.e.*, distension of the renal cavity, may ensue. The symptoms of ureteral calculus, like those of renal calculus, frequently have reference only to the bladder, and the cases become mistaken for cystitis. The acidity of the urine, and the diffusion of pus through the urine even after standing, and the absence of ropy mucus, point to pyelitis as opposed to cystitis. Ureteritis, either of calculous or tuberculous origin, may produce a thickening of the ureter which may be felt at the brim of the pelvis and be mistaken for impacted calculus. A prolapsed and adherent ovary may simulate calculus in the lower end of the ureter. The calculus will, however, be felt more superficially in the vagina, and often through the antero-lateral vaginal wall, below and in front of the cervix, which an ovary never is. The hardness, shape, and direction will also serve to distinguish a calculus from an ovary. Calculi have been felt through the parieties at the brim of the pelvis in rare cases. Through the vagina a calculus can be felt and dealt with in the lower two and a half or three inches of ureter, but through the rectum only in the lower one and a half inches. The symptoms are the same as those in stone in the kidney, and, as in that condition, may be absent. Anuria will result from impaction if the other kidney is not performing its functions, or if the two ureters become blocked simultaneously. Even if the stone escapes in such a case the poisoning effects may have been so severe that death results. Anuria is said to be sometimes produced by a reflex effect upon the other kidney. There may be a hydro-nephrotic tumour, either constant or intermittent, produced by ureteral stone. One of the rarer effects is prolapse of the ureter into the cavity of the bladder, and even, in the female, through the urethral orifice. Among the possible pathological effects are stricture of the ureter, rupture of the ureter above the impaction and entravasation of urine, thickening of the whole length of the ureter or even conversion into a fibrous cord, and discharge of the calculus into the rectum after suppuration. The kidney may be in any stage of hydro or pyo-nephrosis, and fistulæ may form in the loin, or communicating with the stomach, colon, or pleura. The treatment is operation without delay, and removal of the stone, by one of the extra-peritoneal methods of ureterotomy, if it cannot be extracted through the renal pelvis. There is great danger to life in delay if active symptoms continue. Morris thinks the operation will become almost as uniformly successful as nephro-lithotomy. The mode of removal, whether by the loin, groin, sacral, vaginal, pre-rectal, or vesical route, will depend upon the position in the ureter of the impaction. The trans-peritoneal removal is never advised, as the urine is so often septic. The ureter should be incised a little higher than the precise point of impaction, if the stone can be pushed upwards, as its tissues will be there less damaged. If in the vesical part of the ureter the stone should be removed per urethram in the female, or by cystotomy in the male. Dr. P. J. Freyer[5] records two successful cases of operation for impacted ureteral calculus. The first was that of an officer, aged 23, who had suffered for 19 months from renal colic and hæmaturia. At no period did the pain shoot down into the testicle, but passed to the groin. At times he had painless hæmaturia; at other times had much pain, chiefly left-sided. He had passed two stones through the urethra. About a fortnight before the operation he had an acute attack of pain lasting 72 hours, and this time it was in the right side. The right kidney then became enlarged, and was palpable and tender. The kidney was incised through its convex border. A rush of urine occurred. There was no renal stone, but a long probe touched a stone in the ureter four inches from the renal pelvis. The incision was extended, the peritoneum pushed forward, and the stone removed by ureterotomy, as it could not be moved upwards to the kidney. The ureter was not sutured afterwards. Bloody urine was passed the same night, showing patency of the ureter. Free drainage was provided. At the end of six months the patient was in excellent health. The second case was that of a man of 53. He had been a patient at St. Peter's Hospital off and on for 13 years, suffering from multiple stricture, and from symptoms of kidney stone. The left kidney had been explored twice, in 1895 and in 1897, with negative results. In the latter case no kidney could be found. Twelve months after this the patient had great irritability of the bladder, and occasionally the right loin became swollen, and the swelling disappeared with an increase of pus in the urine. The cystoscope now revealed a long, narrow, rough stone, projecting into the bladder from the right ureteral orifice. The stone was caught in the blades of a lithotrite, pulled into the bladder, crushed there, and the débris removed. His health was much improved after this. Freyer[6] refers to five other cases of impacted stone in the ureter which he had operated upon. In three the stones were found in operating upon what were supposed to be ordinary cases of vesical calculus.

[1] Annals of Surgery, Sept., 1899. [2] Ibid. [3] Med. Rec., Nov., 1899. [4] Clin. Jour., Nov., 1899. [5] Lancet, Dec., 1899. [6] Lancet, ii., 1899.

